# Macrophages in ventricular remodeling and heart failure: orchestrators of inflammation and repair

**DOI:** 10.3389/fimmu.2025.1682294

**Published:** 2025-11-19

**Authors:** Han Feng, Wenhui Hu, Ying Liu, Xiaoshuang Xu, Ping Zhou, Chen Qu, Zhengxia Liu

**Affiliations:** 1 Department of Geriatrics, The Second Affiliated Hospital, Nanjing Medical University, Nanjing, Jiangsu, China; 2 Key Laboratory for Aging & Disease, Nanjing Medical University, Nanjing, Jiangsu, China

**Keywords:** cardiac macrophages, ventricular remodeling, heart failure, immune crosstalk, targeted immunotherapy

## Abstract

Heart failure is a complex clinical syndrome caused by structural and/or functional cardiac abnormalities. Ventricular remodeling contributes to its progression. Cardiac macrophages regulate inflammation, fibrosis, and tissue repair that drive this process. In this Review, we describe the origins and phenotypic diversity of cardiac macrophages, including both resident and monocyte-derived subsets. In the left ventricle, macrophages respond to ischemia, pressure overload, and metabolic stress. In the right ventricle, they display distinct immune features under pulmonary hypertension and other stress conditions. We further discuss the interactions between macrophages and other cardiac cell types, such as fibroblasts, cardiomyocytes, endothelial cells, and lymphocytes. These interactions shape the immune environment and structural integrity of the myocardium. We also highlight recent advances in single-cell and spatial technologies that reveal chamber-specific macrophage signatures. Finally, we summarize emerging therapeutic strategies targeting macrophages, including pharmacological agents, engineered cell therapies, and nanoparticle-based delivery systems. Together, these insights provide a framework for understanding macrophage-mediated remodeling and for guiding precision immunotherapies in heart failure.

## 1 Introduction

Heart failure (HF) is a clinical syndrome characterized by structural and/or functional cardiac abnormalities resulting in impaired ventricular filling or ejection, leading to dyspnea, fatigue, and fluid retention. Globally, HF is a major health burden, affecting more than 64 million individuals, with rising prevalence driven by population aging and increased survival from acute cardiovascular events (1, 2). Despite advances in pharmacologic and device-based therapies, hospitalization and mortality rates remain high (3, 4). Ventricular remodeling is characterized by progressive structural and functional alterations in the myocardium and represents a hallmark of various cardiovascular diseases as well as a key determinant of heart failure development and prognosis (5). Initiated by cardiomyocyte death or stress, ventricular remodeling involves inflammatory activation, extracellular matrix (ECM) dysregulation, and progressive fibrosis, ultimately contributing to ventricular dilation, dysfunction, and adverse prognosis (6). Increasing evidence suggests the central role of immune cells in orchestrating these remodeling processes (7). Among them, macrophages have emerged as critical regulators, bridging injury and repair. In addition to their canonical phagocytic function, cardiac macrophages actively modulate the inflammatory environment, coordinate with fibroblasts, endothelial cells, and cardiomyocytes, and influence ECM remodeling and scar formation.

Recent advances in single-cell transcriptomics, lineage tracing, and spatial analysis have revealed a diverse population of cardiac macrophages (8–10). These cells originate from both embryonic progenitors and circulating monocytes, and their phenotypes span a spectrum from pro-inflammatory to reparative states. Their behavior is tightly regulated by local cues, including ischemia, pressure overload, metabolic stress, and neurohormonal signals, all of which vary between the left and right ventricles (11). Accumulating evidence indicates that macrophage-driven remodeling is not uniform across cardiac chambers, with the right ventricle exhibiting distinct immune patterns and responses under stress conditions such as pulmonary hypertension (12).

This review aims to integrate recent findings from basic research and preclinical models to provide a comprehensive overview of the multifaceted roles of macrophages in ventricular remodeling and the progression of heart failure. We first outline the origins and heterogeneity of cardiac macrophages, followed by a comparative analysis of their functional roles and regulatory mechanisms in left versus right ventricular remodeling. We further highlight the dynamic crosstalk between macrophages and other cardiac cell types, including fibroblasts, cardiomyocytes, endothelial cells, and immune cells. Additionally, we evaluate various therapeutic strategies targeting macrophage function, including small-molecule modulators, engineered cell therapies, and nanoparticle-based delivery systems. By integrating mechanistic insights with translational advances, this review seeks to provide a conceptual framework and theoretical foundation for the development of immunomodulatory interventions in ventricular remodeling.

## 2 Origin and heterogeneity of cardiac macrophages

Cardiac macrophages exhibit diversity in both developmental origin and function, populating the heart from early embryogenesis and persisting throughout postnatal life (13). Two major developmental pathways contribute to the cardiac macrophage pool: resident macrophages derived from embryonic progenitors and monocyte-derived macrophages from the bone marrow. Evidence from lineage-tracing and parabiosis studies in murine models has shown that macrophages originating from the yolk sac and fetal liver colonize the developing heart and are maintained through local self-renewal under homeostatic conditions, without continuous input from circulating monocytes (14). These resident macrophages are often identified by the expression of CX3C chemokine receptor 1 (CX3CR1), T-cell immunoglobulin and mucin domain-containing protein 4 (TIMD4), and lymphatic vessel endothelial hyaluronan receptor 1 (LYVE1), and can be subdivided into subpopulations such as TIMD4^+^LYVE1^+^ repair-associated macrophages and MHCII^high^ antigen-presenting macrophages (6). In contrast, bone marrow-derived monocytes are recruited into the myocardium in response to inflammation, ischemia, or mechanical overload, where they undergo differentiation into macrophages whose functions are shaped by the surrounding microenvironment (15, 16).

Based on C-C chemokine receptor 2 (CCR2) expression, cardiac macrophages can be divided into two functionally distinct subsets: CCR2^-^ resident macrophages and CCR2^+^ monocyte-derived macrophages (17). CCR2^-^ macrophages are typically anti-inflammatory and promote tissue repair, angiogenesis, and clearance of apoptotic cells. By comparison, CCR2^+^ macrophages are pro-inflammatory and are major drivers of leukocyte recruitment, cytokine secretion, and fibrotic remodeling (18). In response to different signaling molecules, macrophages can also polarize into functionally distinct phenotypes, namely M1 and M2. Classically activated M1 macrophages are pro-inflammatory and are induced by stimuli such as lipopolysaccharide (LPS) and interferon-γ (IFN-γ), leading to the production of inflammatory cytokines. In contrast, selectively activated M2 macrophages exhibit anti-inflammatory gene expression profiles and reparative phenotypes, contributing to immunomodulation and tissue repair (19). Although macrophages play a crucial role in cardiac remodeling, an imbalance in macrophage polarization between the pro-inflammatory M1 and anti-inflammatory M2 phenotypes may exacerbate inflammation and promote cardiac injury (20).

Recent advances in single-cell RNA sequencing and spatial transcriptomics have revealed unexpected diversity among cardiac macrophages. Subsets such as CD72^hi^ inflammatory macrophages, TIMD4^+^LYVE1^+^ repair macrophages, and TREM2^+^ remodeling-associated macrophages exhibit context- and region-specific distributions (6, 14). Notably, spatial differences in macrophage composition have been observed between the left and right ventricles, suggesting that local microenvironmental factors and mechanical stress gradients may influence macrophage phenotype and function (21, 22). This regional heterogeneity may underlie chamber-specific immune responses in ventricular remodeling, setting the stage for understanding why right ventricular inflammation and fibrosis progress differently from those of the left ventricle.

## 3 Cardiac macrophages and their impact on common heart diseases

Cardiac macrophages shape the initiation, propagation, and resolution of inflammation across major cardiac diseases. This section outlines disease-specific responses and highlights chamber-specific features when supported by data.

### 3.1 Myocardial infarction

Myocardial infarction (MI) triggers a dynamic and phase-specific immune response, in which macrophages play indispensable roles throughout injury, inflammation, and repair. During the early phase of MI (days 1-3), the infarcted myocardium is rapidly infiltrated by Ly6C2^hi^ CCR2^+^ monocytes from the circulation, which differentiate into pro-inflammatory macrophages (10). These CCR2^+^ macrophages release inflammatory cytokines such as IL-1β and TNF-α, and contribute to necrotic tissue clearance. Meanwhile, the pool of tissue-resident CCR2^-^ macrophages, which are prenatally seeded and self-renewing in homeostasis, declines due to ischemic injury (23). From day 3 onward, a reparative shift begins to emerge: CCR2^+^ macrophages gradually transition into anti-inflammatory phenotypes, including Trem2^hi^, Spp1^+^, and Legumain^+^ subsets. Single-cell RNA sequencing and fate-mapping studies have confirmed a sequential differentiation trajectory from monocytes to inflammatory macrophages and ultimately to reparative Trem2^hi^ macrophages. Trem2^hi^ macrophages exhibit high expression of IL-10, TGF-β, and osteopontin (Spp1). *In vivo* administration of these cells was shown to promote scar maturation and improve remodeling (10, 24). Macrophage metabolism also shapes post-MI outcomes: CCR2^+^ subsets favor glycolysis via HIF-1α activation, while reparative macrophages shift toward oxidative phosphorylation and fatty acid oxidation (25). While most clinical and experimental data emphasize left ventricular involvement, right ventricular injury can also occur; therefore, we discuss macrophage responses in MI in a chamber-agnostic manner, noting chamber-specific features when supported by evidence.

### 3.2 Pressure overload and hypertrophy

Hemodynamic pressure overload, as observed in clinical settings such as hypertension or aortic valve stenosis, induces concentric hypertrophy and a persistent, low-grade inflammatory response. In preclinical models like transverse aortic constriction (TAC), myocardial infiltration of immune cells, especially macrophages, is a defining feature of maladaptive cardiac remodeling under sustained mechanical stress (26). Single-cell transcriptomic analysis in TAC-treated mice has revealed the emergence of functionally distinct macrophage subsets during hypertrophy. An early-appearing population of CD72^+^ CCR2^+^ macrophages expresses high levels of pro-inflammatory and pro-fibrotic genes (TNF, IL-1β, IL-6), suggesting involvement in oxidative stress regulation and immune activation (27). By contrast, CCR2^-^ resident macrophages represent a tissue-resident population that is replenished locally through *in situ* proliferation and shows minimal contribution from circulating monocytes. These cells are thought to perform distinct homeostatic and remodeling functions, contrasting the inflammatory profile of recruited CCR2^+^ macrophages (27, 28). Additional evidence shows that CX3CR1^+^ resident macrophages contribute to maintaining capillary integrity and limiting fibrotic expansion in pressure overload hearts. Depletion of CX3CR1^+^ cells impairs angiogenesis and exacerbates interstitial fibrosis in TAC models (26). Beyond the TAC model, recent evidence from hypertensive heart failure with preserved ejection fraction (HFpEF) models shows that CXCR4^+^ macrophages promote myocardial fibrosis by repressing peroxisome proliferator-activated receptor γ (PPARγ) activity and upregulating chemokine(C-X-C motif) ligand 3(CXCL3), which in turn drives myofibroblast differentiation through the CXCR2 pathway (29). Altogether, these findings reveal a functional divergence between monocyte-derived inflammatory macrophages and resident reparative macrophages in pressure overload-induced cardiac remodeling. The relative dominance of each subset may influence the progression toward adaptive versus maladaptive hypertrophy.

### 3.3 Metabolic or adrenergic stress

Metabolic and adrenergic stress reprogram cardiac immune landscapes toward pro-inflammatory and pro-fibrotic states, with macrophages acting as key effectors (21, 30). In models of obesity-induced cardiomyopathy, macrophages expressing doublecortin-like kinase 1 (DCLK1) accumulated in the LV and promoted pathological remodeling by activating the RIP2-TAK1 signaling cascade. This axis amplifies cytokine release and fibroblast activation, leading to extracellular matrix deposition and hypertrophy. Inhibition of DCLK1, either genetically or pharmacologically, significantly attenuates these adverse effects in high-fat diet (HFD)-fed mice, highlighting DCLK1 as a metabolic sensor of macrophage-mediated remodeling (31). Similarly, in diabetic hearts, the absence of the neuropeptide substance P (SP) shifts the macrophage population toward a pro-inflammatory M1 phenotype, characterized by increased expression of TNF-α and IL-1β and reduced IL-10 production. SP replacement therapy restores the balance toward M2 macrophages, thereby reducing myocardial fibrosis and preserving diastolic function (32). Adrenergic signaling also drives maladaptive remodeling through macrophage-dependent inflammatory activation. In isoproterenol (ISO)-induced heart failure models, myeloid differentiation factor 2 (MD2) was significantly upregulated in both cardiac macrophages and cardiomyocytes. MD2 activation is triggered via the β-adrenergic receptor-cAMP-PKA-ROS signaling cascade, which amplifies proinflammatory responses and promotes myocardial injury (33). In parallel, a distinct mechanism involving the ovarian tumour deubiquitinase 1 (OTUD1)-caspase-associated recruitment domain 9 (CARD9) axis has been identified in ISO-challenged hearts. Under these conditions, OTUD1 deubiquitinates CARD9, facilitating the assembly of the CARD9-BCL10-MALT1 (CBM) signaling complex in macrophages. This promotes NF-κB activation and the expression of proinflammatory cytokines. Mice lacking OTUD1 or CARD9 exhibit reduced macrophage activation and are protected from ISO-induced structural and functional cardiac remodeling (34).

In addition to neurohormonal stimuli, dyslipidemia exerts profound immunometabolic effects on the cardiac environment. In a model of hyperlipidemia-induced diastolic dysfunction, single-cell RNA sequencing of *ApoE* knockout mice fed a Western diet revealed marked expansion of multiple macrophage subsets. These included pro-inflammatory CCR2^+^ cells and lipid-stressed Timd4^+^ resident macrophages. Metabolic overload, particularly through long-chain saturated fatty acids like laurate and myristate, triggered endoplasmic reticulum stress and upregulation of inflammatory genes such as IL-1β and CXCL10. These activated macrophages were spatially associated with fibrotic regions and contributed to a maladaptive cardiac response through paracrine signaling to cardiomyocytes, involving TNF-α, IL-6, and IL-15. Collectively, these findings define a macrophage-mediated immunometabolic axis linking lipid accumulation to inflammatory and fibrotic remodeling (35). Collectively, these studies demonstrate that metabolic and adrenergic stressors converge on macrophage-driven signaling cascades to orchestrate inflammatory and fibrotic remodeling.

### 3.4 Pulmonary arterial hypertension and arrhythmogenic right ventricular cardiomyopathy

Pulmonary arterial hypertension (PAH) imposes chronic pressure stress on the right ventricle (RV), leading over time to maladaptive hypertrophy, interstitial fibrosis, and eventual contractile failure (36, 37). Compared to the left ventricle, the RV exhibits greater susceptibility to inflammation-driven remodeling under these conditions. Emerging evidence suggests that macrophages play a crucial role in orchestrating this pathological process. In mice and rat PAH models, such as monocrotaline (MCT), Sugen-hypoxia (SuHx), and hypoxia-only (Hyp), researchers found a clear rise in RV macrophage infiltration, especially those derived from circulating monocytes. Some of these express CCR2 and are linked to inflammation and structural damage. Among key inflammatory pathways, the NLRP3 (nucleotide-binding domain, leucine-rich-containing family, pyrin domain-containing-3) inflammasome stands out (38). In PAH models, RV macrophages show high levels of NLRP3, which activate caspase-1 to mature IL-1β. RV tissues in PAH patients confirm increased NLRP3 and IL-1β compared to non-PAH or LV samples, indicating a chamber-specific inflammatory pattern (38, 39). In addition, caspase-8 has been implicated in non-canonical inflammasome signaling in macrophages. Elevated caspase-8 activity supports IL-1β maturation and enhances inflammatory polarization during PAH progression, although its exact contribution to RV remodeling requires further investigation (40). Macrophages are proposed to participate in extracellular matrix remodeling during RV failure. In PAH models, inflammatory macrophage subsets increase in number and upregulate profibrotic mediators such as IL-1β and MMP9 (38). While CCR2^+^ macrophages dominate the RV immune landscape under pressure overload, direct spatial evidence linking these cells to fibrotic regions remains limited and warrants further investigation.

Recent findings in arrhythmogenic right ventricular cardiomyopathy (ARVC) further expand the pathogenic roles of macrophages in RV diseases. In myocardial and fibro-fatty tissues of ARVC patients, pro-inflammatory CCL3^+^ macrophages accumulate and produce high levels of IL-1β and TNF-α, correlating with disease severity and heart failure progression. These macrophages likely contribute to localized immune activation, fibrotic remodeling, and arrhythmic risk through paracrine cytokine signaling and interactions with cardiomyocytes and lipid-laden stromal cells. Their presence reinforces the view that macrophage-driven inflammation in the RV is not limited to pressure-overload conditions like PAH but may be a shared mechanism in diverse RV pathologies (41, 42). Together, these findings suggest that macrophages not only reflect the inflammatory state of the RV but also drive structural changes and contractile impairment in multiple right heart diseases. Their chamber-specific behavior makes them promising targets for immunomodulatory interventions.

### 3.5 Pulmonary artery banding

**Figure 1:**
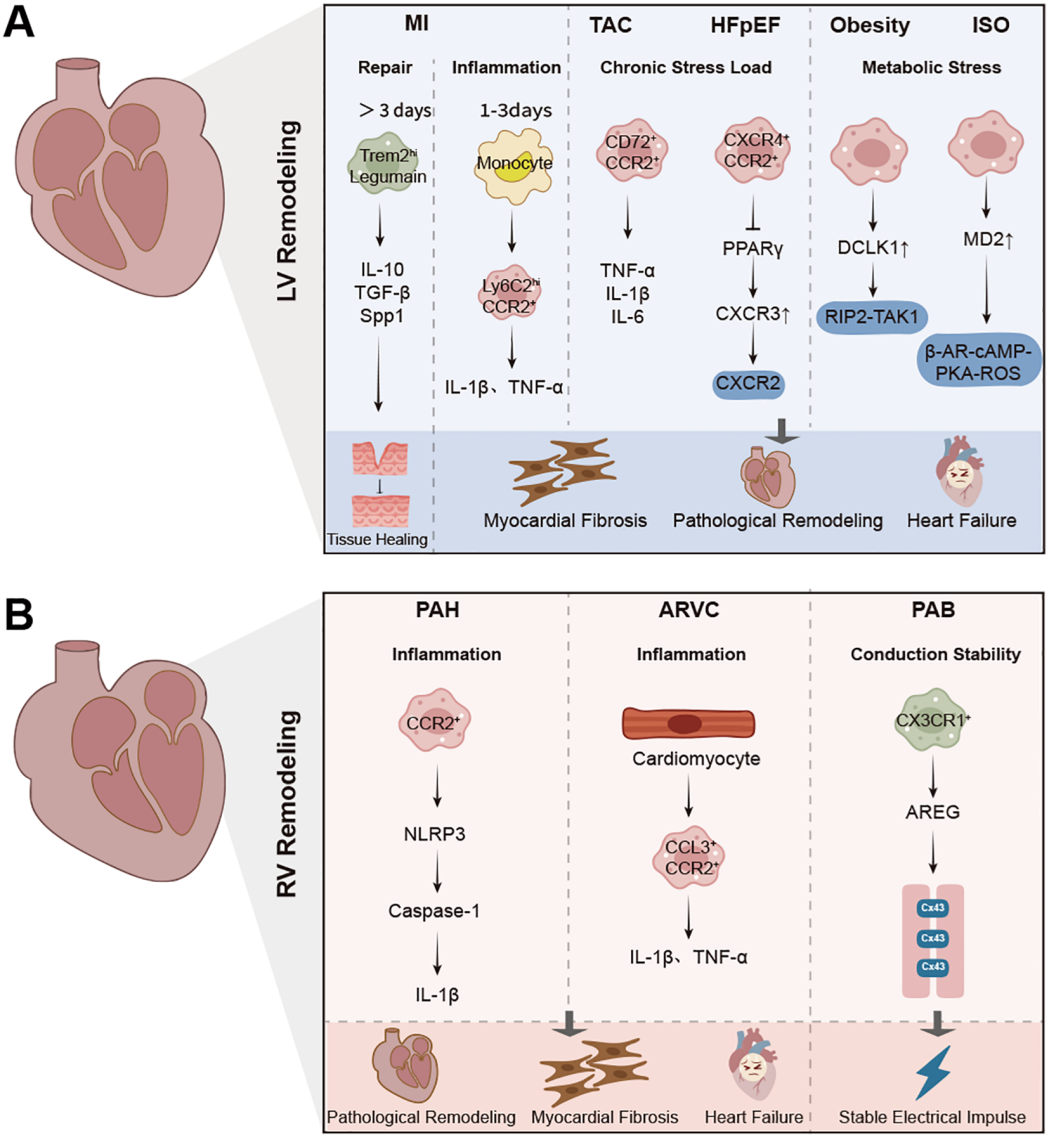
Macrophage-mediated inflammation and remodeling in left and right ventricular diseases. **(A)** Schematic representation of macrophage phenotypes, signaling pathways, and pathological outcomes in left ventricular (LV) remodeling, including myocardial infarction (MI), transverse aortic constriction (TAC), hypertensive heart failure with preserved ejection fraction (HFpEF), and obesity models. Acute macrophage activation is characterized by Ly6C^hi^ CCR2^+^ monocyte recruitment, inflammation (IL-1β, TNF-α), and fibroblast activation. Reparative macrophages (Trem2^hi^, Legumain^+^) promote tissue repair, angiogenesis, and scar maturation through IL-10, TGF-β, and Spp1. Chronic stress and metabolic stress induce CCR2^+^, CD72^+^, and CXCR4^+^ macrophages that drive fibrosis via TNF-α, IL-6, and PPARγ pathways. **(B)** Overview of macrophage subsets, signaling mechanisms, and outcomes in right ventricular (RV) remodeling, including pulmonary arterial hypertension (PAH), arrhythmogenic right ventricular cardiomyopathy (ARVC), and pulmonary artery banding (PAB) models. CCR2^+^ macrophages are recruited in response to pressure overload, promoting fibrosis and inflammation via NLRP3 inflammasome and IL-1β. CX3CR1^+^ macrophages, associated with conduction stability, secrete amphiregulin (AREG) to stabilize Connexin43. The pathogenesis of RV failure is characterized by macrophage-driven fibrosis, arrhythmia, and maladaptive remodeling.

Not all macrophage activity in the RV contributes to injury. Under specific conditions, especially during early or isolated mechanical stress, resident macrophages provide structural and functional support to the RV, helping to preserve homeostasis and prevent sudden decompensation. One prominent example is the role of CX3CR1^+^ tissue-resident macrophages in maintaining electrical conduction. In the pulmonary artery banding (PAB) model, selective depletion of macrophages results in a sharp increase in arrhythmic death. Mechanistic studies show that CX3CR1^+^ macrophages produce amphiregulin (AREG), which helps organize connexin 43 in gap junctions. This macrophage-cardiomyocyte interaction stabilizes impulse propagation and protects against conduction failure during RV stress (15). In addition to their role in conduction, macrophages may also contribute to vascular remodeling in the RV. In chronic hypoxia-induced pulmonary hypertension models, researchers have observed significant increases in capillary length, volume, and density in the RV as early as 7 days after the onset of pressure overload (43). Although not all studies directly trace this to macrophages, prior evidence indicates that resident cardiac macrophages can produce vascular endothelial growth factor A(VEGF-A), Wnt ligands, and other angiogenic factors that support endothelial cell proliferation and microvascular expansion (26, 44). These vascular adaptations may help maintain oxygen delivery and delay RV decompensation. A third protective mechanism involves the buffering of oxidative stress. Macrophages possess antioxidant defense programs and can reprogram their metabolism under stress. This includes upregulation of glutathione pathways, mitochondrial quality control, and scavenging of reactive oxygen species (ROS) (45, 46). While most of these data derive from studies in the LV, the presence of resident macrophages with similar transcriptional signatures in the RV suggests a conserved anti-oxidative role. Taken together, these findings support the idea that resident macrophages in the RV are not simply bystanders or pro-inflammatory mediators. Instead, they act as immunoregulatory sentinels that support conduction integrity, vascular adaptation, and redox balance during RV stress. Distinguishing these protective subsets from inflammatory CCR2^+^ monocyte-derived macrophages may provide new targets for preserving RV function in disease. Comparative analyses of left and right ventricular diseases highlight distinct macrophage phenotypes, signaling pathways, and pathological outcomes, as summarized in recent models of ventricular remodeling (**Figure 1**) and the major macrophage subsets, functional roles, and key mechanisms across LV and RV pathologies (**Table 1**).

**Table 1:** Functional roles of macrophages in distinct ventricular diseases.

Disease model/etiology	Affected ventricle	Major macrophage subtypes	Functional direction	Key mechanisms or factors	References
MI	LV/RV	CCR2^+^, Ly6C^hi^, Trem2^hi^	Inflammation+Repair	IL-1β, TGF-β, phagocytosis, angiogenesis	(10, 23)
TAC	LV	CCR2^+^, TIMD4^+^/CCR2^-^, CD72^+^	Pro-fibrotic/Protective	TNF, IL-1β, oxidative stress modulation	(27)
HFpEF	LV	CXCR4^+^	Pro-fibrotic	PPARγ, CXCL3	(29)
HFD/Diabetes	LV	DCLK1^+^, M1, Timd4^+^	Metabolic sensing/Pro-fibrotic	RIP2–TAK1, TNF-α, IL-6, CXCL10	(31, 32, 35)
ISO-induced cardiomyopathy	LV	MD2^+^, CARD9^+^	Pro-inflammatory/Pro-fibrotic	β1-AR-cAMP-PKA-ROS, NF-κB	(34)
PAH	RV	CCR2^+^, Caspase-8^+^	Inflammation/ECM remodeling	NLRP3, IL-1β, MMP9, TGF-β	(38, 40)
ARVC	RV	CCL3^+^、CD68^+^	Pro-inflammatory/Pro-fibrotic	IL-1β, TNF-α, fibro-fatty infiltration	(41)
PAB	RV	CX3CR1^+^	Electrical stability/Protective	AREG–EGFR, Cx43	(15)

MI predominantly involves the LV, with RV involvement reported in a subset of cases.

## 4 Crosstalk with other cardiac cells

### 4.1 Cardiac fibroblasts

Cardiac fibroblasts are key mediators of ECM homeostasis and fibrotic remodeling. Their interaction with both resident and recruited macrophages has emerged as a central axis in determining the balance between adaptive repair and pathological fibrosis in the stressed heart. In the context of MI, macrophages infiltrate the infarcted myocardium and secrete a range of fibrogenic signals, including TGF-β1, IL-1β, TNF, and Spp1, which promote fibroblast activation and differentiation into collagen-producing myofibroblasts. Single-cell transcriptomic studies have revealed that scar-associated Spp1^+^ macrophages interact directly with reparative cardiac fibroblasts (RCFs) via ligand-receptor pairs such as Spp1-CD44 and TGFB1-TGFBR2, contributing to ECM deposition and infarct maturation (47). This macrophage-fibroblast coupling forms a transient pro-fibrotic niche, especially during the early phase after injury. In a mouse model of HFpEF, macrophages contributed to diastolic dysfunction by promoting fibroblast activation through CXCL12-CXCR4 signaling. Macrophage-specific CXCL12 deletion or fibroblast-specific CXCR4 knockout both attenuated ECM accumulation and improved ventricular compliance, demonstrating that this macrophage-fibroblast signaling loop actively drives fibrotic remodeling (29, 48). Pressure overload models (such as TAC) further confirm that macrophage subsets can shape fibroblast responses. Recruited CCR2^+^ monocyte-derived macrophages promote fibrosis via IL-10 and TGF-β secretion, while resident CX3CR1^+^ macrophages support a reparative response and limit fibroblast overactivation (26, 49). Activated fibroblasts produce cytokines and chemokines, including granulocyte-macrophage colony-stimulating factor (GM-CSF), chemokine (C-C motif) ligand 2 (CCL2), and others that contribute to immune cell recruitment and macrophage activation. These signals create a feedforward inflammatory loop that amplifies immune responses and promotes fibrosis (6).

RV remodeling in response to pressure overload exhibits distinct ECM features compared with the LV. Experimental and clinical studies of PAH have demonstrated that inflammation is typically pronounced in the RV, with marked infiltration of macrophages into the myocardium. These immune cells release a spectrum of pro-fibrotic cytokines and growth factors that may directly stimulate fibroblast activation and ECM production. In parallel, pressure overload triggers fibroblast expansion and their transition into a myofibroblast phenotype, characterized by elevated synthesis of type I collagen and other matrix components. The resulting fibrosis in the RV is typically distributed in both interstitial and perivascular regions and contributes to diastolic dysfunction and progressive stiffening of the ventricular wall. Although resident macrophages in the LV have been implicated in modulating fibrotic responses, such regulatory mechanisms have not been clearly defined in the RV setting (50, 51). Overall, the interplay between fibroblasts and macrophages is dynamic, context-specific, and central to the structural remodeling of both ventricles. While the LV benefits from resident macrophages that temper fibroblast activation, the RV’s reliance on recruited inflammatory macrophages may exacerbate fibrosis and compromise function in disease states.

### 4.2 Cardiomyocytes

Cardiomyocytes are both sources and targets of macrophage-mediated signaling during cardiac injury and remodeling. Following MI, necrotic cardiomyocytes release damage-associated molecular patterns (DAMPs), including ATP, high mobility group box-1 (HMGB1), and mitochondrial DNA. These signals activate Toll-like receptors and inflammasome pathways in cardiac macrophages, especially NLRP3 and cyclic GMP-AMP synthase (cGAS)-stimulator of interferon genes (STING), and initiate a potent inflammatory response. This process enhances the secretion of IL-1β and TNF-α, which contribute to immune cell recruitment and tissue clearance (50, 52). Macrophages not only respond to cardiomyocyte-derived signals but also regulate cardiomyocyte fate and function. In the early phase post-MI, macrophage-mediated efferocytosis clears dying cardiomyocytes and apoptotic neutrophils, thereby promoting resolution of inflammation and transition to repair. The receptor MerTK (mer proto-oncogene tyrosine kinase), expressed by reparative macrophages, is essential for apoptotic cell clearance and for limiting adverse remodeling (53, 54). Beyond inflammation resolution, macrophages participate in cardiomyocyte quality control. Recent evidence shows that cardiomyocytes extrude damaged mitochondria via structures called exophers, which are then engulfed and degraded by neighboring macrophages through lysosomal pathways. This process is essential for maintaining mitochondrial homeostasis and preventing cardiomyocyte dysfunction under stress conditions such as pressure overload or ischemia (55). Notably, the nature of macrophage-cardiomyocyte crosstalk may vary between the left and right ventricles. While most studies focus on the LV post-infarction, emerging data suggest that in RV pressure overload, cardiomyocyte stress may contribute to paracrine macrophage activation through neurohumoral and oxidative mechanisms. However, direct experimental studies dissecting RV-specific macrophage-cardiomyocyte interactions remain limited (50).

### 4.3 Endothelial cells

The interaction between macrophages and endothelial cells (ECs) is essential for shaping the inflammatory response, vascular remodeling, and tissue repair following MI and pressure overload. Endothelial cells serve as both sensors and regulators of myocardial injury, and they dynamically influence macrophage recruitment, phenotype, and function. In the acute phase of MI, injured endothelial cells upregulate adhesion molecules and secrete chemokines such as CCL2, which promote the recruitment of CCR2^+^ monocytes into the infarcted myocardium (56). These monocytes differentiate into macrophages that contribute to both inflammation and repair (57). Macrophages influence endothelial function by secreting proangiogenic factors, including vascular endothelial growth factor (VEGF) and interleukins such as IL-4 and IL-6, which enhance endothelial tube formation and support neovascularization in the injured region (58). Reparative macrophages also contribute to maintaining endothelial integrity during the cardiac repair phase. These macrophages secrete TGF-β, which promotes endothelial cell survival and helps stabilize the vasculature by reducing permeability (59). However, if inflammatory signaling persists or oxidative stress remains elevated, endothelial dysfunction may occur, leading to capillary rarefaction and impaired tissue perfusion, especially under pressure overload conditions (57). This vulnerability is more pronounced in RV, where endothelial cells appear more susceptible to stress and less capable of compensatory angiogenesis (50). Additionally, macrophage-derived TGF-β can induce endothelial-to-mesenchymal transition (EndoMT) through the MT1-MMP/TGF-β/Smad2 signaling axis, thereby contributing to fibrotic remodeling. Inhibition of this pathway has been shown to reduce collagen deposition and improve microvascular architecture in experimental models (60).

### 4.4 Other immune cells

In the normal heart, the immune landscape is dominated by resident macrophages distributed within the interstitium and perivascular niches. Small numbers of dendritic cells (DCs), B cells, regulatory T cells (Tregs), natural killer (NK) cells/innate lymphoid cells (ILCs), and mast cells are also present, whereas inflammatory monocytes and neutrophils are typically absent at baseline. DCs, which develop under Flt3L and enter the myocardium via CCR2-dependent cues, sample self-antigens and help sustain peripheral tolerance by licensing Tregs; consistent with this, MHC II^+^cardiac antigen-presenting cells can process α-myosin heavy-chain peptides and activate T cells ex vivo (61). In turn, Tregs provide IL-10-centered anti-inflammatory signals and modulate monocyte-to-macrophage differentiation, supporting resident macrophage programs of efferocytosis, immune surveillance, and matrix homeostasis (62). Collectively, resident macrophages act as central organizers of cardiac immunity, engaging DCs, T cells (including Tregs), B cells, and NK/ILCs through antigen presentation and paracrine mediators, whereas mast cells and granulocytes are sparse at baseline and participate predominantly during injury (6).

During myocardial infarction, neutrophils rapidly infiltrate and communicate bidirectionally with macrophages: neutrophil-derived DAMPs and cytokines amplify macrophage recruitment, whereas macrophage efferocytosis curtails neutrophil lifespan and limits collateral injury (63). Regulatory T cells (Tregs) interact with macrophages to promote resolution of inflammation and healing after MI. Tregs inhibit pro-inflammatory macrophage activation and enhance efferocytosis (64). They secrete IL-35, which not only promotes CCR2^-^ MHC-II^low^ macrophage survival but also enhances their expression of TGF-β1, thus fostering an anti-inflammatory environment (65). Notably, macrophage-derived CCL17 competes with CCL22 and suppresses Treg recruitment, worsening inflammation in the infarcted myocardium (66). Together, these interactions highlight that macrophages act not only as effectors of innate immunity but also as central modulators of adaptive immune dynamics during cardiac injury and repair.

Under pressure overload, macrophage and T-cell crosstalk becomes a key determinant of remodeling: Th1/Th17-skewed signals favor inflammatory CCR2^+^ macrophages and fibrosis, whereas Treg-derived mediators promote reparative programs. Mast cells can augment fibroblast activation and matrix deposition, while DCs help sustain chronic low-grade T-cell activation. In HFpEF-like conditions, metabolic stress further biases macrophages via chemokine axes that are reinforced by lymphocyte-derived cues (11).

In PAH and ARVC, macrophages engage T cells, dendritic cells, and mast cells to potentiate inflammasome activation, pro-inflammatory cytokine production, and fibro-fatty remodeling. By contrast, under isolated mechanical stress such as pulmonary artery banding, resident macrophages cooperate with non-macrophage immune populations to preserve electrical conduction integrity, promote microvascular adaptation, and maintain redox homeostasis, underscoring that immune–macrophage crosstalk can be protective in a context-dependent manner (67).

## 5 Therapeutic targeting of macrophages

### 5.1 Pharmacological modulation

Modulating macrophage behavior through pharmacological agents represents a promising strategy for altering the course of cardiac injury and remodeling. Several molecular targets, such as CCR2, IL-10, STAT3, and specific metabolic pathways, have been investigated in preclinical studies. One key axis involves CCR2^+^ monocyte recruitment, which drives early inflammation after MI or pressure overload. IL-34, acting via the NF-κB pathway, has been shown to promote macrophage infiltration and polarization toward a pro-inflammatory phenotype, thereby exacerbating ischemia-reperfusion injury. Pharmacological inhibition of this signaling axis, such as using CCR2 antagonists or IL-34 neutralization, has been proposed to limit acute inflammation and reduce infarct size (68). Polarization strategies have garnered significant interest due to their capacity to reshape macrophage behavior during cardiac injury. Anti-inflammatory cytokines such as IL-10 and IL-4 are well-established inducers of reparative M2 polarization. In preclinical models of myocardial infarction, quercitrin administration improved cardiac remodeling by promoting M2-like macrophage polarization, characterized by upregulation of Arg1, Ym1, and Mrc1, and by enhancing mitochondrial oxidative metabolism and reducing intracellular ROS levels (69). Additionally, activation of the STAT3 pathway has been shown to expand MerTK^+^ reparative macrophages, which facilitate efferocytosis and support tissue healing (11). Nevertheless, excessive activation of anti-inflammatory programs may impair timely immune clearance and extracellular matrix remodeling, highlighting the need for precise modulation of macrophage function. Other targets include MD2, a co-receptor for TLR4. Chronic β-adrenergic stimulation, such as by isoproterenol, upregulates MD2 via the β-AR-Camp-PKA-ROS signaling axis, aggravating inflammatory heart failure. Inhibiting MD2 alleviates both macrophage activation and cardiomyocyte damage (33). Transcriptional and metabolic regulators also influence macrophage behavior in the injured heart. Macrophage-specific deletion of yes-associated protein (YAP) and transcriptional co-activator with PDZ-binding motif (TAZ), two core effectors of the Hippo signaling pathway, has been shown to shift macrophage phenotype toward a reparative profile. In a mouse model of myocardial infarction, YAP/TAZ deficiency reduced pro-inflammatory cytokines such as TNF-α and IL-6 while increasing Arg1 expression, ultimately improving infarct healing and cardiac function (70). Similarly, knockout of doublecortin-like kinase 1 (DCLK1) in macrophages attenuated RIP2/TAK1 signaling, suppressing inflammatory macrophage activation and protecting against obesity-induced cardiomyopathy (31). Pharmacological modulation must balance pro-repair and anti-inflammatory functions. Excessive inhibition of immune pathways, particularly during the early inflammatory phase, may impair debris clearance and proper scar formation. Thus, timing and context are essential considerations for therapeutic success (71).

### 5.2 Cellular or engineered macrophage therapy

Cell-based approaches offer a direct means to reprogram cardiac inflammation and promote myocardial repair through macrophage manipulation. These strategies include adoptive cell transfer, ex vivo reprogramming, and genetic engineering of macrophages or their modulators. One strategy involves the transplantation of reparative macrophages. For example, infusion of legumain-overexpressing cardiac resident macrophages significantly enhanced clearance of apoptotic cardiomyocytes after MI, contributing to reduced infarct size and improved cardiac function. Mechanistically, this therapeutic effect is largely dependent on the activation of LC3-associated phagocytosis (LAP), which serves as a key downstream target of legumain to promote efficient phagolysosomal degradation of apoptotic material (24). This finding emphasizes the benefit of leveraging endogenous reparative properties through engineered macrophage-based therapy. Another avenue is the development of CAR-M (chimeric antigen receptor-macrophage) platforms. In a recent study, CAR-Ms targeting fibroblast activation protein (FAP) were administered in ischemia-reperfusion (I/R) models. These FAP CAR-Ms efficiently infiltrated infarcted myocardium, phagocytosed activated fibroblasts, and ultimately reduced myocardial fibrosis and improved cardiac function. The therapy conferred both acute and long-term cardioprotection, highlighting its potential in resolving post-I/R remodeling (72).

In addition to direct macrophage engineering, manipulating Tregs can modulate macrophage phenotypes *in vivo*. Systemic delivery of exogenous Tregs following MI promoted cardiac repair by reducing pro-inflammatory Ly6C^+^CCR2^+^ macrophages and promoting their transition toward a reparative profile (73). This shift was mediated in part by IL-10 and nidogen-1, indicating that Tregs serve as upstream regulators of macrophage behavior in the injured myocardium. MicroRNA-based modulation has also emerged as a cell-level strategy. In a chronic heart failure (CHF) model, inhibition of microRNA-21-3p (miR-21-3p) reduced excessive mitophagy in cardiomyocytes and suppressed M1 macrophage polarization. The miR-21-3p inhibitor reversed L-palmitoyl carnitine–induced cardiotoxicity by restoring carnitine palmitoyltransferase 1A (CPT1A) expression and dampening pro-inflammatory responses in co-cultured macrophages, suggesting an indirect method to reprogram immune metabolism (74). Despite their promise, cellular therapies face challenges. These include potential immune rejection, low survival or engraftment rates of transferred cells, and difficulties in achieving targeted homing to the injured myocardium. Moreover, macrophage plasticity *in vivo* may dilute the effects of ex vivo programming. Nonetheless, these approaches represent an important translational frontier, offering multi-modal control over cardiac inflammation and tissue remodeling.

### 5.3 Nanoparticles and targeted delivery strategies

Targeted nanoparticle systems offer a promising avenue for enhancing macrophage-specific modulation in cardiac repair. One notable strategy involves mannan-functionalized metal-organic framework nanoparticles (Que@MOF/Man), designed for the targeted delivery of quercetin to inflamed myocardium. These particles preferentially accumulate in recruited macrophages via mannose receptor-mediated uptake, alleviating oxidative stress and promoting M2-like polarization, which indirectly improves cardiomyocyte viability through intercellular crosstalk (75). Similarly, cardiac-resident macrophage-derived extracellular vesicles (EVs) modified with monocyte membranes have been engineered to deliver thymosin β4 (Tβ4) specifically to infarcted tissue. This biomimetic coating enhances homing efficiency and promotes neovascularization and cardiomyocyte survival via Akt and integrin-linked kinase signaling pathways (76). Another approach leverages inflammation-targeted nanomedicine to both scavenge reactive oxygen species and reprogram macrophage metabolism. *In vivo*, studies show that these platforms markedly improve left ventricular function and reduce post-MI fibrosis (75). Beyond nanoparticles, endogenous factors such as myeloid-derived growth factor (Mydgf) have been shown to activate regenerative programs. Delivered systemically or via direct myocardial injection, Mydgf enhances cardiomyocyte proliferation through the c-Myc/FoxM1 axis, representing a paracrine strategy to stimulate heart regeneration with macrophage involvement (77). These delivery strategies enable cell-specific, timely, and synergistic control of macrophage phenotypes, highlighting their translational potential in reversing maladaptive remodeling. These delivery platforms enable cell-specific, timely, and synergistic modulation of macrophage phenotypes, offering translational potential to reverse maladaptive ventricular remodeling. Collectively, pharmacological agents, engineered macrophage therapies, and targeted nanomedicines provide complementary strategies to regulate inflammatory signaling, promote reparative polarization, and modulate intercellular crosstalk in LV and RV diseases (**Table 2**).

**Table 2:** Potential therapeutic strategies targeting cardiac macrophages.

Intervention strategy	Mechanism	Target/Pathway	Disease model	Therapeutic effect	Reference
CCR2 antagonist	reduce the accumulation of pro-inflammatory macrophages	CCL2-CCR2	MI, pressure overload	↓ acute inflammation, ↓ infarct size	(68)
IL-10 therapy	Promotes M2-like macrophages	IL-10, STAT3	MI	↑ repair, ↓ fibrosis	(11, 69)
MD2 inhibitor	Inhibits β-AR–PKA–ROS signaling	MD2–TLR4	Inflammatory HF	↓ macrophage activation, ↓ cardiomyocyte damage	(33)
YAP/TAZ knockout	Reprograms macrophage phenotype	Hippo pathway, IL-6/TNF-α	MI	↑ Arg1, ↑ healing	(70)
Legumain-overexpressing macrophages	Enhances apoptotic cell clearance	LAP	MI	↓ infarct size, ↑ function	(24)
CAR-M therapy	Targets fibroblasts, enhances efferocytosis	FAP, IL-1β/TNF-α	I/R injury	↓ fibrosis, ↑ function	(72)
miR-21-3p inhibitor	Reduces mitophagy, reprograms metabolism	CPT1A, miR-21-3p	CHF	↓ M1 polarization, ↑ homeostasis	(74)
Que@MOF/Man nanoparticle	Promotes M2 via mannose receptor	Oxidative stress, M2 markers	MI	↑ viability, ↓ ROS	(75)
Tβ4-EV w/monocyte membrane	Enhances homing, angiogenesis	Akt, ILK	MI	↑ vascularization, ↑ survival	(76)
Mydgf protein	Stimulates regeneration via paracrine effect	c-Myc/FoxM1	Neonatal heart	↑ cardiomyocyte proliferation	(77)

↑ = increase/upregulation/improvement; ↓ = decrease/downregulation/reduction.

## 6 Emerging technologies and future directions

### 6.1 New tools and translational platforms for macrophage-targeted cardiac therapy

Recent advances in single-cell and spatial technologies have dramatically expanded our understanding of cardiac macrophage diversity. Single-cell RNA sequencing (scRNA-seq) and spatial transcriptomics have identified region-specific macrophage subsets in the infarcted heart, such as Bhlhe41^+^ cells, which display distinct spatial localization and fibroblast-suppressive functions (78). These tools have revealed that macrophage behavior and gene expression profiles vary not only between cell types but also across time points post-injury. Fate-mapping strategies and multi-omics platforms such as CITE-seq and lineage tracing have further clarified the origin and turnover of CCR2^+^ monocyte-derived macrophages versus yolk sac-derived resident subsets, with implications for therapeutic targeting (79). Multiomic profiling has also uncovered chromatin accessibility differences and enhancer usage patterns that distinguish reparative from inflammatory macrophage states (80). These insights lay the foundation for precise manipulation of macrophage subpopulations. Building on this mechanistic knowledge, several engineered platforms are under development to reprogram macrophage function *in situ*. For example, CAR-M has been successfully used to target FAP in myocardial ischemia-reperfusion injury, promoting antifibrotic effects and functional recovery (72). Other strategies include engineered EVs loaded with reparative peptides like thymosin β4, modified with monocyte-derived membranes to enhance cardiac tropism (76). Stimuli-responsive nanoparticles and heart-on-a-chip platforms further extend delivery precision and allow for evaluation of human-derived immune-cardiac interactions *in vitro (*81). These emerging technologies offer new avenues to rewire macrophage phenotype and activity in a context-dependent and cell-specific manner.

Despite encouraging preclinical results, translating macrophage-targeted therapies into the clinic faces multiple hurdles. Engineered constructs such as CAR-Ms and nanocarriers require validation in large-animal models to assess safety, biodistribution, and immunogenicity. Even in macrophage-rich settings such as the infarcted heart, drug or cell delivery remains inefficient due to immune clearance or off-target uptake. Moreover, macrophage plasticity *in vivo* raises concerns about phenotypic stability after administration. Lessons can be drawn from the limited success of CCR2 inhibitors in clinical trials and from ongoing challenges in CAR-T therapy, where cytokine release syndrome and antigen escape constrain utility. As such, scalable manufacturing, precise delivery, and functional durability must be carefully addressed to harness the full translational potential of macrophage-modulating platforms (71, 82, 83).

### 6.2 Major challenges and future directions

Despite remarkable progress in defining the heterogeneity and functions of cardiac macrophages, several critical knowledge gaps and major challenges remain. A key unresolved issue concerns the spatial and temporal dynamics of macrophage-fibroblast and macrophage-cardiomyocyte interactions, which are still incompletely understood, particularly in RV and in non-ischemic etiologies. While macrophage function in LV remodeling has been extensively investigated, RV-specific immunobiology remains poorly characterized. Emerging studies indicate that RV macrophages differ from their LV counterparts in ontogeny, polarization potential, and cytokine responsiveness (38, 84). Species differences between murine and human cardiac macrophages further complicate translation, as most datasets are derived from mice and lack comprehensive spatial or single-cell characterization in human hearts. Integrating spatial multi-omics and *in vivo* lineage-tracing approaches will be crucial to delineate chamber-specific macrophage niches and identify actionable molecular targets for translational research.

Another important direction concerns the intercellular communication networks that shape cardiac inflammation and remodeling. Although macrophage interactions with cardiomyocytes and fibroblasts are well documented, their crosstalk with non-classical cardiac cell types such as neurons, adipocytes, and lymphatic endothelial cells remains poorly defined. Recent findings indicate that neuroimmune and metabolic signaling circuits strongly influence macrophage activation and recruitment. For example, perivascular adipose tissue secretes adipokines that modulate macrophage infiltration, whereas sympathetic nerve activity dynamically regulates macrophage-driven remodeling under stress conditions (79, 85, 86). Elucidating these multidimensional interactions may reveal actionable therapeutic targets for cardiac immunomodulation. In addition, existing macrophage-targeted therapies often lack subset or chamber specificity and may inadvertently suppress reparative macrophage populations. Future studies should therefore focus on developing precision immunomodulatory strategies that selectively modulate pathogenic subsets while preserving protective and reparative functions to prevent maladaptive remodeling and heart failure progression.

Standardizing macrophage nomenclature and functional classification also remains an urgent priority. The conventional M1/M2 paradigm oversimplifies the diversity of cardiac macrophages revealed by single-cell studies (87). Future classification frameworks should integrate spatial localization, ontogeny, metabolic state, and transcriptomic characteristics to establish a more comprehensive taxonomy. In this context, advances in machine learning (ML) and artificial intelligence (AI) offer powerful tools to deconvolute high-dimensional datasets. Integrating ML algorithms with multi-omic data may facilitate target prediction, subtype classification, and response modeling, thus accelerating precision immunotherapy (88). In conclusion, answering these unresolved questions will not only deepen our mechanistic understanding but also expand the therapeutic landscape for macrophage-directed strategies in cardiovascular disease.

## 7 Conclusion

Cardiac macrophages play essential roles in the initiation, amplification, and resolution of inflammation during ventricular remodeling. Their phenotypic and functional diversity reflects distinct developmental origins and environmental cues. While resident macrophages contribute to homeostasis and repair, recruited subsets often drive pathological remodeling. The balance between these macrophage subsets determines the pattern and extent of cardiac structural remodeling. Accumulating evidence indicates chamber-specific immune programs, particularly the underexplored macrophage phenotypes in the right ventricle. However, key questions remain regarding the spatial dynamics, temporal transitions, and intercellular signaling mechanisms that govern macrophage behavior in different pathological contexts. Recent advances in single-cell and spatial transcriptomics provide powerful tools to map macrophage function *in situ*. Future studies should focus on identifying macrophage subtypes with therapeutic potential and uncovering key molecular targets that regulate their phenotypic transitions. A deeper understanding of macrophage biology will support the design of immunotherapies that prevent adverse remodeling while preserving essential immune functions.
